# Effectiveness of Educational Practices in University Students’ Knowledge about Sun Protection and Its Relation to Sunlight Exposure: An Exploratory Study in a Portuguese Higher Education Institution

**DOI:** 10.3390/ejihpe10030053

**Published:** 2020-07-15

**Authors:** Bárbara Roque Ferreira, João Simões, Maria Eduarda Ferreira

**Affiliations:** 1 Department of Dermatology, Centre Hospitalier de Mouscron, Centre for Philosophy of Science of the University of Lisbon, 1749-016 Lisbon, Portugal; barbara.roqueferreira@gmail.com; 2 Department of Otorhinolaryngology (ENT)/Head and Neck Surgery, Centre Hospitalier de Mouscron, Faculty of Medicine, University of Coimbra, 3004-354 Coimbra, Portugal; jofsim@gmail.com; 3 Higher School of Education, Communication and Sport, CI&DEI, Polytechnic Institute of Guarda, Research Unit for Inland Development, Polytechnic of Guarda, 6300-559 Guarda, Portugal

**Keywords:** skin health, skin neoplasms, sunlight, knowledge, practices, students

## Abstract

Nowadays, there is worldwide recognition that health and educational outcomes are inextricably linked. It is also recognized that health education comprises opportunities to improve health literacy, including the improvement of knowledge and the development of life skills to promote individual health. It is also known that the behavioral practices regarding sun exposure are an important risk factor for skin cancer. Research is needed in this area to understand the contribution of the “Education for Health” curricular unit to these issues. Our exploratory research sought to collect information about the knowledge and practices regarding sun exposure of a group of Portuguese university students who have already attended this curricular unit. The results indicate that the participants show that, notwithstanding that they have already attended this curricular unit, they do not have more literacy on skin health, do not perceive that sun exposure habits are related to skin health and do not perceive that photoprotection constitutes prevention of skin cancer. The results support the need to promote the necessary reflection and debate on the way in which health education should be taught, as well as what is taught, in order to empower students to get decision-making skills associated with the adoption of healthier attitudes and practices, thus helping to prevent skin cancer.

## 1. Introduction

Since skin is a well-adapted barrier of the human body with important functions [1], including defending the body from aggressions induced by ultraviolet solar radiation, it is the behavior that compromises this adaptation, namely the corporal exposure to solar radiation during long periods, over time and without any type of photoprotection. Sun exposure is a well-known risk factor in the etiopathogenesis of melanoma and non-melanoma skin cancer. The way in which each individual manages their functional reserve into the homeostatic biological balance throughout their life cycle depends upon the options of the individual, i.e., it is related to behaviors. This is a main factor for health condition, taking into account that “Health is a dynamic state of wellbeing characterized by a physical, mental and social potential, which satisfies the demands of life commensurate with age, culture, and personal responsibility” [2] (p. 336). Furthermore, the Ottawa Charter [3] states that effective health promotion leads to changes in health determinants. The determinants include both those that people control (behaviors, lifestyles, use of health services), and those beyond their control (socio-economic and environmental conditions as well as the provision of services) [4]. Higher education institutions with statutory attributions for the development of activities in the education field, which aim at the initial and postgraduate levels to cultivate professional, scientific, technical and pedagogical-didactic aspects, will certainly play an important role regarding health promotion for their students.

This educational research attempts to understand if the “Education for Health” curricular unit has contributed to improving knowledge and health practices about sun exposure of their students. Our research set out to collect the information described above with a group of Portuguese university students who have already attended the Education for Health curricular unit, included in the curricula of the educational courses.

### Problem and Objectives

The research question was: does the “Education for Health” curricular unit contribute to improving knowledge on sun exposure and sunlight protection of the students? This research question intends to understand the effectiveness of the educational practices on sun protection knowledge and their relation to healthy practices on sunlight exposure. In addition, the question also aims to collect information on the knowledge and practices concerning solar exposure, and to contribute to the debate and reflection on the effectiveness of educational practices’ role in improving students’ skills on skin health related to sun exposure.

## 2. Related Work

The importance of behavior for the health of individuals is now consensual in the scientific community, with several studies on the relationships among behavior, health and several pathologies [5]. In fact, behavior is a modifiable health determinant [6]. There is a strong scientific interest regarding sun protection and risk behaviors, because of the increase in the prevalence of skin cancer. One of the most popular sun protection measures is the use of sunscreen. It has been demonstrated that deleterious effects on the skin from excessive sun exposure are cumulative [7] and it is estimated that six out of 10 cases of skin cancer are related to excessive sun exposure [8]. Non-melanoma skin cancer, and specifically basal cell carcinoma and squamous cell carcinoma, are the most common cancers in the Caucasian race and scientific literature suggests that “the incidence of this type of cancer could be prevented if individuals adopted precautionary behaviors” [9] (p. 37). In 2018, in Portugal, new cases of non-melanoma skin cancer were ranked seventh and new cases of cutaneous melanoma were ranked thirteenth when compared to other countries worldwide [10]. Exposure to ultraviolet radiation is considered a major etiological factor correlated with the increase of the incidence of these neoplasias [11,12]. Cutaneous melanoma is the most aggressive and lethal type of all skin cancer, resulting from the malignant transformation of melanocytes related to genetic defects most often caused by ultraviolet radiation. It corresponds to approximately 5% of all skin cancers, but to three-quarters of all deaths due to skin cancer. Furthermore, it has a relevant prevalence and increasing incidence, particularly over the last 50 years. In Western countries, it is strongly correlated with skin color, the presence of freckles and the number and characteristics of naevi as well as with behaviors, such as past sun exposure during childhood and adolescence, specifically when it has been intense and intermittent [12,13].

The Surgeon General and World Health Organization have presented guidelines to improve sun protection in order to address the increasing incidence of skin cancer. These guidelines reinforce the importance of behaviors in this prevention, consisting of wearing protective clothing, sunglasses and a hat, the importance of seeking shade and avoiding sun exposure during peak sunlight hours and the central relevance of sunscreen. Educational interventions can change intentional decision-making processes by increasing one’s knowledge and improving socio-cognitive determinants, such as the attitudes and learning of skills needed to perform adequate behavior [14]. “All health educators, regardless of work setting, will find the path to change by scanning for and thinking about new opportunities” [15] (p. 269).

Although existing health services in the community are efficient, disease prevention and health promotion will always be conditioned by the appropriation of healthy behavioral practices and routines, that is, the appropriation of competences for autonomous decision-making in relation to health needs (self-empowerment based on health literacy). Health literacy is a global issue [16] that has implications that are far-reaching and impact both the individual and society [17]. Furthermore, health literacy, disadvantage and risk for poorer health outcomes are correlated [18], even though it is unclear to what extent health literacy may affect health outcomes [19]. Indeed, health literacy encompasses “health knowledge, beliefs and practices, capacity and self-efficacy, community empowerment” [20] (p. 16). It seems that the development of health literacy is the way to construct adequate responses that enable individuals to control health determinants and therefore to adopt everyday practices that enhance and benefit their biological functional reserve and, thus, their health. Therefore, initiatives may focus on educational practices as a means to achieve the improvement of the knowledge and the development of life skills that are conducive to individual health. The construction of the biological functional reserve of each individual is built into early adult life. Then, health or risk behaviors in this period of the life cycle will influence, through a positive or negative manner, the next phases of this cycle. Thus, health-promoting interventions in this age group will have medium- and long-term consequences [21,22,23].

It seems necessary for individuals to “understand their health potential, their own health determinants and specificities associated with their life cycle and context stage, and develop knowledge, attitudes, competencies and accountability that promote health and prevent disease concerning themselves, their families, communities and their environments” [24] (p. 64). There is increasing recognition that health and educational outcomes are inextricably linked [25]. Health promotion is the “empowerment of people and communities to change the determinants of health for one’s own quality of life” [3]. Therefore, there is a need to understand the role of higher education in promoting youth health, particularly regarding the knowledge about sun protection and its relation to sunlight exposure.

## 3. Research Methodology

This exploratory research was framed within the Education for Health curricular unit, involving a total of 94 participants. The selection criteria applied to participants were: a group of the students (n = 47) who had already attended the “Education for Health” curricular unit (study group) and another group of the students (n = 47) that had not attended the curricular unit (control group).

The “Education for Health” is a curricular unit included in the curricula of educational courses (degree in Basic Education, an undergraduate degree in monitoring of children and youths) of a public Higher Education Institution with more than 40 years of work in the formation of educators and teachers in the field of education. The students of the Masters in Pre-School and Primary School Teaching are former students of the course on Basic Education, so they had already attended that curricular unit. These students participated on a voluntary basis. The learning survey was filled out right after the end of the classes of the curricular unit.

Reading the program description of the “Education for Health” curricular unit of the educational courses of this public Portuguese higher education institution, we note that the general objectives are: to understand that health is a result of a biological balance and that prevention is the best way to keep it; to learn about health risk factors; to raise awareness of health promotion; to explore pedagogical tools for the design and implementation of health education projects and actions in educational contexts; to discuss health education interventions in scientific and interdisciplinary contexts; to develop research skills in the field; to promote reflection and self-analysis potentially oriented towards problem-solving and decision making; and to evaluate different educational methods and techniques to promote healthy practices.

The plan of the teaching and learning process of the “Skin and sun exposure (sun protection: benefits, risks and healthy practices)” content of the curricular unit is presented in Table 1. This content was taught in two lessons (one week). An integrated method that comprises a lecture (1 h) followed by a discussion section was applied. In the discursive interaction, the professor is a mediator and instigator.

A learning survey was developed to collect data. The questions were formulated considering the construction of questionnaires of the same type with special attention to the structure and order of the questions [26,27,28,29,30] and it was based on the learning contents of “Skin and sun exposure (sun protection: benefits, risks and healthy practices)” that are taught in the curricular unit “Education for Health”. The questions of the learning survey were specifically designed for this study by a dermatologist doctor, based on their clinical background and on a literature review including scholarly articles, reviews and original research related to the affects of individual behaviors regarding sun exposure [28,29,30]. It is an anonymous self-reported learning survey [8,9,12,14,15] to collect information about knowledge and practices concerning solar exposure from the students who have already attended the “Education for Health” curricular unit. The learning survey was distributed to twenty students from the same institution (not included in the study). No difficulties of interpretation were recorded, and the experts considered the final version suitable to apply in the study group and in the control group. Thus, the questions were agreed upon by a consensus process of the research team. More precisely, the final version included 25 questions, including multiple-choice, closed-ended and open-ended questions, addressing the knowledge about benefits and risks of sun exposure: practices of photoprotection and knowledge about the relevance of photoprotection. An example of the multiple-choice and the closed-ended questions that were used is: “Do you like sunlight exposure during the warmer summer months?” (Answer: yes or no) If “yes”, “In what period of the day?” (Answers: (a) before 10 AM; (b) between 10 AM and 4 PM; (c) after 4 PM; (d) before 10 AM and after 4 PM). An additional example of a closed-ended question and of an open-ended question that were used: “Do you know the harmful effects of the sun exposure on the skin?”; “If you have answered “yes” in the previous question, please mention some examples of the harmful effects of the sun exposure on the skin”. Another example of the open-ended questions is: “Please mention some example of the benefits of sunscreens”.

Information about the age, sex and skin phototype of the participant was also included in the analysis of the results. The skin phototype was obtained through the identification of the participants of one of six possibilities concerning their skin color and the response to sun exposure, taking into account a simplified version of the traditional Fitzpatrick phototype. For example, for phototype II: color of the skin—white; skin characteristics—always burns, tans slightly or sensitive to the sun. No difficulties in the interpretation were noted by the students who were not included in the study and who answered to the questions before the study group. The work meets the standards of ethics established for this type of study at the authors’ institution. It follows the ethical guidelines of the current Declaration of Helsinki.

The data were entered into IBM Statistical Package for the Social Sciences (SPSS) Statistics Version 20 (IB). The X2 test was used to analyze proportions between groups and comparison of continuous variables between groups was performed with the Student’s t-test. Statistical significance was set at 0.05.

## 4. Results and Discussion

The participation rate on this learning survey was 71.4%. No difficulties in the interpretation of the questions were reported by the participants.

The study groups had a mean age of 20.9 ± 2.2 years, including two male and 45 female participants (Table 2).

The control group consisted of individuals with a mean age of 20.6 ± 2.6 years, with two male and 45 female participants (*p* > 0.05).

In the descriptive analysis of the learning survey, it was verified that the predominant Fitzpatrick phototypes (Table 1) were II and III, and there was no statistical significance between the two groups (*p* > 0.05). The Fitzpatrick scale is based on a person’s skin color and sun sensitivity (burning and tanning). Fitzpatrick skin typing helps predict the risk of photodamage and skin cancer [31]. The skin types I–III are at an increased risk of sun damage and skin cancer, particularly cutaneous manifestations of photoaging, melanoma and non-melanoma skin cancer [32].

**Figure 1:**
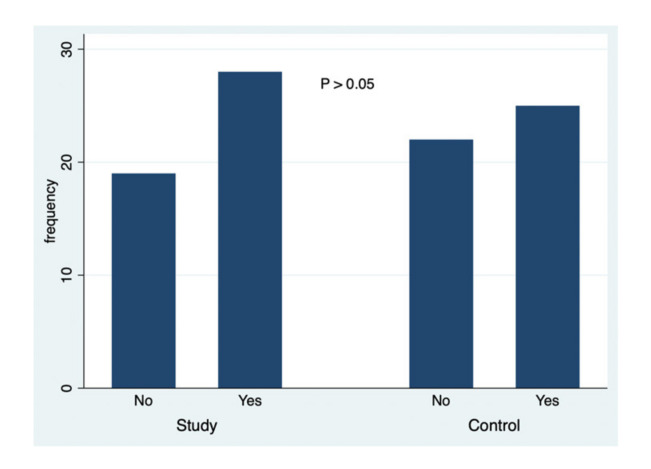
The responses to the question: Do you know the beneficial effects of sun exposure?

**Figure 2:**
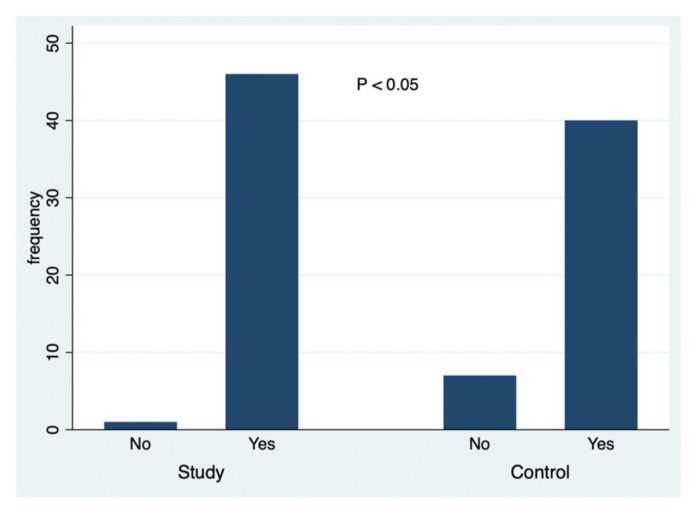
The responses to the question: Do you know the damages (harmful effects) on our skin due to sun exposure?

**Figure 3:**
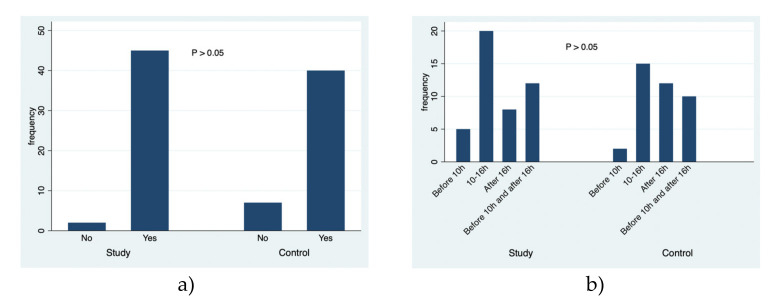
The responses to the questions: (**a**) Do you like to expose yourself to the sun on the beach during the summertime? (**b**) If your answer is “yes”, please state when do you expose yourself to the sun during the summertime?

**Figure 4:**
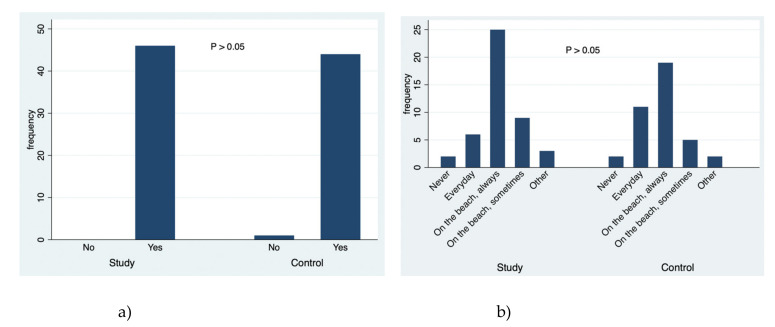
The responses to the questions: (**a**) Do you consider the use of sunscreen important? (**b**) When should one apply the sunscreen?

**Figure 5:**
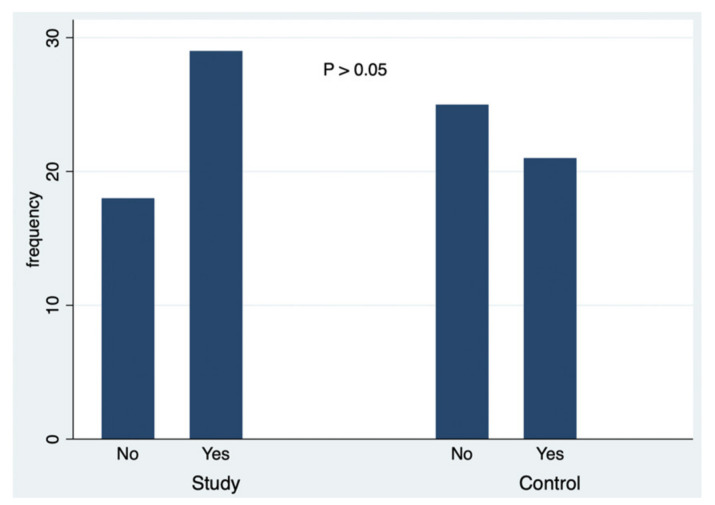
The responses to the question: Were you already informed about photoprotection (measures to reduce exposure to the sun)?

**Figure 6:**
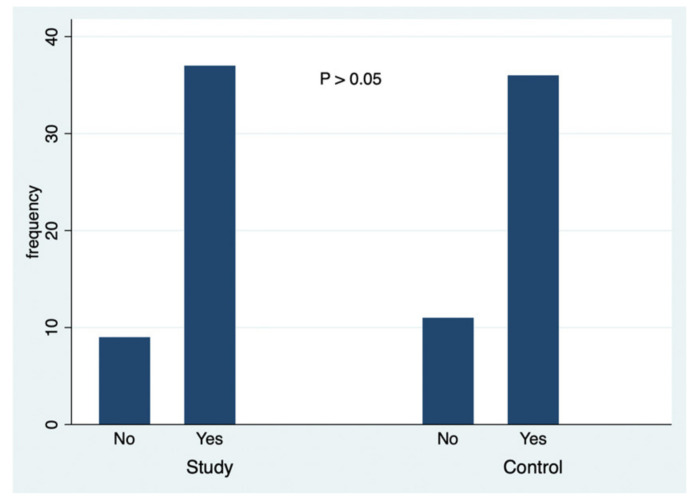
The responses to the question: Have you ever had a sunburn?

A simultaneous analysis was made about the data collected in this study, and Figure 1, Figure 2, Figure 3, Figure 4, Figure 5 and Figure 6 illustrate some of the questions that were used in this learning survey and the statistical analysis of the responses that were given by the participants.

Globally, the analysis of the responses (Figure 1) and their comparison between groups (study and control groups) showed that there was no significant association between being a student of courses in the area of education and the knowledge of the beneficial effects of sun exposure (*p* = 0.533). The total number of respondents who knew about the beneficial effects of sun exposure was higher (28 students); however, there was no statistical significance in relation to students who reported that they did not know the beneficial effects (19 students).

For the open-ended question “If you answered yes in the previous question, cite examples of the beneficial effects of sun exposure”, the predominant response was the production of vitamin D due to sun exposure, with statistical significance. Indeed, the body obtains vitamin D by cutaneous synthesis upon skin sunlight exposure and vitamin D has been implicated in several health benefits [33]. However, although the exposure to ultraviolet radiation in sunlight is related to the obtaining of vitamin D, this radiation is known to be related to a higher risk of skin cancer in later life [34,35]. At any rate, this was the only correct answer that was given, and all other responses were incorrect examples of the beneficial effects of sun exposure. There was no statistically significant difference between the responses of the study and the control groups (*p* = 0.805).

However, the analysis of the question “Do you know the damages (harmful effects) on our skin due to sun exposure?” (Figure 2) showed that there was a statistically significant association between being a student of education and having noticed the harmful effects of excessive sun exposure (*p* = 0.027).

However, there was no statistically significant difference between the two groups with respect to the examples given for the harmful effects of excessive sun exposure (*p* = 0.384) and there was no significant difference between the study and control groups (Figure 3) regarding the pleasure associated with sun exposure, during the summertime, at the beach (*p* = 0.08). Moreover, when the participants were asked to say when they expose themselves to the sun, both groups answered between 10 AM and 4 PM (Figure 3b, *p* = 0.463).

Regarding the relation between skin cancer and sunburn, 19 participants agreed with it and 11 students denied it in the study group, while 20 agreed with it and 4 students denied it in the control group. In the past several decades, there has been concern about sun exposure and sun protective measures because of the increasing incidence of skin cancer [23]. Although the responses provided were correct (skin cancer, sunburn or aging), the students belonging to the study group did not remember more than one example and there was no tendency with statistical significance among the responses that were given (*p* = 0.08). Some studies have noted that it is not always the best knowledge levels that entail higher sun protection practices and lower sunburn incidence rates [36,37]. Regarding examples of beneficial effects from the use of sunscreen, there was no association between more knowledge about the subject and belonging to one of the groups (*p* = 0.122).

The majority of students incorrectly pointed out “skin hydration” as a beneficial effect of sunscreen (22 students from the study group and 24 from the control group); 11 students from the study group and 6 from the control group reported the prevention of burns, and only six students from the study group and four from the control group mentioned the prevention of cutaneous cancer. It should be noted that most skin cancers could have been prevented with protection from exposure to sunlight [38].

Regarding the knowledge about the relevance of the use of sunscreens (Figure 4a), most of the students from both the study and control groups perceived the use of sunscreen to be important, with no statistically significant difference between them (*p* = 0.309). Regarding how much time should the sunscreen be applied before sun exposure, the study group selected the answer “15 min before sun exposure”, which is the correct response, with statistical significance in relation to the control group (*p* = 0.006); 26 students from the study group chose the “15 min earlier” answer and in the control group 18 students chose the “15 min earlier” answer. Concerning the question about when sun exposure should be avoided, there was no statistical significance between groups and between the different responses (*p* = 0.0787). Students in both groups tended to correctly answer, “sun exposure should be avoided between 10 AM and 4 PM regardless of whether we use sunscreen”. Thus, there is no concordance between what they consider as correct (avoid sun exposure between 10 AM and 4 PM) and what they usually do (Figure 3b, they expose themselves to the sun between 10 AM and 4 PM). In addition, both groups answered that sunscreen application should be done every day (Figure 4b) during the summertime at the beach (with statistical significance in relation to other responses), and there was no statistical significance compared to the control group even though the correct answer would be every day when one is outside (*p* = 0.123).

Regarding the source of information used to choose the sunscreen (Figure 5), the majority of participants did not seek information, although there was no statistically significant difference between the groups (*p* = 0.0079) and between the different responses. Statistical significance was not found in relation to the control group (*p* = 0.0121) regarding the question of having already received information on photoprotection. Although the number of students in the study group reporting to have received information on this was higher (29 students) than those who said they did not receive information (18), the difference was not statistically significant. There was no statistically significant association between the phototype of each student and the tendency to obtain more information about photoprotection (*p* = 0.365).

The practice of solar exposure showed that there was no association between the study group and the control group. Moreover, regarding the practice of sun exposure at the beach (*p* = 0.080), 45 students of the study group and 40 of the control group reported they like sun exposure in the hot months; however, there was no statistically significant difference between them. Regarding the usual time of sun exposure on the beach, there was no statistically significant difference between the different responses and between the groups (*p* = 0.463).

Regarding the sun protection factor (SPF), there was no statistically significant difference among the different responses and between the groups (*p* = 0.729) and the answer was correct: SPF ≥ 30. Concerning the question about who should use sunscreen daily, there was also no statistically significant difference between the groups (*p* = 0.330) and between the responses. A statistically significant higher number of students answered that all people (infants, children and adults) should use sunscreen daily. The correct answer was “all the people from 6 months of age”. In the last SPF item, most respondents correctly answered that “sunscreen application should be renewed every 2 h”, but there was no statistically significant difference between the type of responses and each group (*p* = 0.593). The SPF provides strong protection against the development of skin cancer [39] and sunscreen application should be renewed every 2 h [40]. In response to the question “Have you ever had a sunburn?” (Figure 6) 37 students from the study group and 36 from the control group reported to have already had a sunburn; thus, there is no evidence of health behaviors in the study group (*p* = 0.652), which is surprising as both groups answered that sunscreen was important, without differences (*p* = 0.309).

The analysis of this question showed a higher tendency, with statistical significance for phototypes II, III and IV, to have a history of sunburn (*p* = 0.002). Although people know the sun-related risks and protection measures, they still do not have healthier practices regarding sun exposure and the rates of the sunburn incidence are high [37].

Interestingly, regarding behavioral practices in relation to sun exposure, no statistical significance was found between the study group and the control group, although the former answered that they were aware of the harmful effects of sun exposure, with a different statistical significance. However, the results showed that the low level of this knowledge has no significant difference between the groups. The results demonstrate that there is an overall tendency of no statistical significance of being a student of higher education courses and having deep knowledge on the relation between sun exposure behavioral practices and the promotion of skin health and prevention of skin cancer. Students in both groups did not demonstrate to have lack of knowledge about the beneficial effects of sun exposure and the use of sunscreen on the skin. Most students, regardless of whether or not they belong to an education course, did not seek information about photoprotection. However, research has shown that deeper knowledge does not necessarily associate with adopting healthier attitudes and behaviors. There is a strong tendency to reject concepts that do not correspond to our prior conceptions [41,42]. Education is needed to empower pupils to carry out sun protection in real life. An educational process is needed, using methods in order to empower students for decision-making skills associated with adopting healthier attitudes and behaviors [35].

These results have shown gaps in the students’ knowledge and their relation to healthy practices on sun exposure. The teaching–learning process of the “Skin and sun exposure (sun protection: benefits, risks and healthy practices)” content of the program of the curricular unit requires reflection, discussion and debate. Assessing coherence between the learning objectives, teaching methodologies and the learning outcomes is, perhaps, the path to follow. Thus, it is necessary to point out that “the educational process is a challenge. It needs to be renewed permanently. Therefore, it is necessary to constantly debate that subject” [43] (p. 1). The results clearly show that there is no association between being a student attending the “Education for Health” curricular unit and: having more literacy on skin health; deeply understanding that sun exposure behavioral practices are related to skin health; having the awareness that photoprotection is a skin cancer prevention practice; and having better behaviors regarding sun exposure. Research that analyzes students’ knowledge about sunlight protection and risks can help identify education problems linked to the teaching–learning process [44]. Studies have drawn attention to the need for the promotion of photoprotective habits and the need for engaging physicians and teachers with this subject [45]. Through targeted educational interventions, higher awareness and knowledge levels could be achieved, as well as the adoption of healthier attitudes and behaviors regarding sun exposure, which would take us to a lower risk for the development of skin cancer [25].

The results of this study highlight the complex interrelationship between knowledge and behavioral health practices. However, if the behavior change process is “any activity that you initiate to help modify your thinking, feeling, or behavior” [46] (p. 25), the “Education for Health” curricular unit will and should play an important role. People acquire a coherent set of experiences, with associations, concepts, values, feelings and conditioned responses that influence their lifestyle. There is a strong tendency to reject concepts that do not correspond to our prior conceptions [29,30]. However, teachers and educators are expected to know and confront epistemological, social and psychological conceptions of beliefs, feelings and behaviors, as well as to evaluate the consequences of these conceptions in the construction and maximization of functional reserves that determine health. Thus, it is a challenge for the teaching–learning process to provide students with life skills about determinants of health in order to improve behavioral health practices.

## 5. Conclusions, Limitations and Recommendations

We may conclude that the study group had gaps concerning the knowledge and the appropriation of skills in relation to behavioral practices regarding sun exposure, highlighting the need for more training regarding behavioral practices and the knowledge of risk in relation to solar exposure. The results of this research provide valuable information to improve knowledge and develop life skills that are conducive to the skin health of the students attending the “Education for Health” curricular unit. The “Education for Health” subject has an important role to play in the development of health literacy. Reflection on teaching methodology to address this issue should be considered, which should also involve epistemological decisions. This study can be taken as an example of the need to develop research on the outcomes regarding the lectures provided by professors who teach these topics. Teaching is not just sharing and transferring knowledge, but it requires reflection to promote intervention and research about educative practices in order to promote healthier behavioral practices. There is increasing recognition that health and educational outcomes are inextricably linked. Promoting health is also the task of higher education schools.

The results of this survey demonstrate the potential of this type of research as an instrument to understand the need to improve knowledge and healthy practices. Moreover, the research shows the need to rethink the curricular unit, namely the methods applied and identifying expectations of the students. This educational research demonstrates the importance of looking into the effects of educational practices through a “learning survey”. Further research is needed to reinforce these results. Nevertheless, this study has some limitations. The questions were not used before, which limits their application and interpretation in other comparable groups. This work might be followed by a project of validation on a larger group of participants. We will have to question the value that these students are attributing to the knowledge of this determinant of health (sun exposure) and the elaboration of a similar study for each one of the determinants of health would be relevant. We believe that research in this field should be based on the intersection of knowledge among specialists in the areas of education and health, and this is the reason why we have decided to conduct a study in the field of education with a multidisciplinary team, including a dermatologist.

The most important insight gained through the present study was that curriculum and educational issues related to empowerment in the health of students need analysis, reflection and discussion. Globally, the results have shown that a deeper knowledge of skin health promotion and skin cancer prevention does not necessarily associate with adopting healthier attitudes and behaviors. This highlights the conclusions of previous studies, that although educational interventions may have positive effects, it is important to remember that the behavior can also be automatically triggered by and deeply linked with environmental characteristics (the family and the social environment), that should be considered in the educational interventions [14]. Therefore, the results support the need to promote the necessary reflection and debate on the way in which health education should be taught and analyzed, taking into account its central role and relevance to improve behaviors and the impact this may have on health promotion and on the prevention of disease. Education may help contribute to improving skin health as affected by sun exposure, helping to encourage the adoption of the correct practices to contribute to preventing the most common (basal cell carcinoma and squamous cell carcinoma) and the most lethal (melanoma) types of skin cancer [12,14]. Therefore, this study provides information for clinicians and educators on the scale of the problem.

## Figures and Tables

**Table 1:** Plan of the teaching and learning process.

Learning Objectives	Teaching Strategies
Understand health as a result of a biological balance and that prevention behaviors are the best way to preserve it.	1st Lesson (2 h) and 2nd Lesson (3 h): Integrated method (Lecture–Discussion)
Explain the benefits (vitamin D) and risks (skin cancer, sunburn or aging) of sun exposure.	
Know healthy practices concerning sun exposure.	
Identify and provide examples of precautions regarding sun exposure.	
Understand the importance of sunscreen and how and when to apply it.	

**Table 2:** Sample characteristics.

Characteristics (Age, Gender and Phototype)	Study Group (n = 47)	Control Group (n = 47)	*p*-Value
Age (mean ± standard deviation, years)	20.9 ± 2.2	20.6 ± 2.6	0.543
Gender (n, number of students Female Male	45 2	45 2	1
Phototype (n, number of students) I II III IV V VI ?*	3 11 16 12 1 0 4	2 17 15 10 0 2 1	0.370

* Unknown phototype (not indicated by the student).
